# Technical validation of controlled exposure to cat dander in the specialized particulate control environmental exposure unit (SPaC-EEU)

**DOI:** 10.1186/s13223-024-00928-1

**Published:** 2025-01-28

**Authors:** Lubnaa Hossenbaccus, Terry Walker, Anne K. Ellis

**Affiliations:** 1 Allergy Research Unit, Kingston Health Sciences Center - KGH Site, Kingston, ON Canada; 2 Department of Medicine, Queen’s University, Kingston, ON Canada; 3 Department of Biomedical and Molecular Sciences, Queen’s University, Kingston, ON Canada

**Keywords:** Allergic rhinitis, Environmental exposure unit, Technical Validation, Controlled allergen challenge facility, Air sampling, Fel d 1, Cat dander, Allergy

To the Editor,

The Environmental Exposure Unit (EEU) is a 3500 square feet controlled allergen exposure facility located at the Kingston General Hospital site of Kingston Health Science Centre –in Kingston, Canada, which has been operating in this location since 1995. It is an internationally recognized model of prolonged allergen exposure, that can seat up to 120 participants at one time, with control of variables such as temperature, humidity, air flow, CO_2_ levels, and allergen type and concentration [1, 2]. The EEU is used to study mechanisms of, and treatments for allergic rhinitis (AR) and has been employed extensively with ragweed, grass, and birch pollens [3–6]. 

For the study of perennial allergens, the Specialized Particulate Control Environmental Exposure Unit (SPaC-EEU; formerly known as the house dust mite (HDM)-EEU) has been established. This is an enclosed 760 sq. ft micro-controlled room within the EEU proper, and includes an adjacent anteroom, designed to hold between 5 and 45 participants simultaneously. It has undergone technical and clinical validation for use with HDM, successfully eliciting AR symptoms and biologic changes in only HDM-allergic, and not non-allergic, participants [7, 8]. 

As accurate and reproducible clinical models of prolonged cat allergen exposure are limited, we sought to evaluate the SPaC-EEU for use with cat dander [9]. We hypothesized that the SPaC-EEU and its equipment are suitable for the accurate and reproducible distribution of cat allergen over time.

We sourced cat dander (Greer^®^, USA) with confirmed concentrations of *Felis domesticus allergen 1* (Fel d 1), the most common cat allergen protein. When assessed with light and scanning electron microscopy, it was evident that the allergen could not easily be visually counted (Fig. 1A). As a result, a Fel d 1-specific enzyme-linked immunosorbent assay (ELISA; Indoor Biotechnologies, USA) was subsequently employed to quantify Fel d 1 concentrations in air samples.

**Fig. 1:**
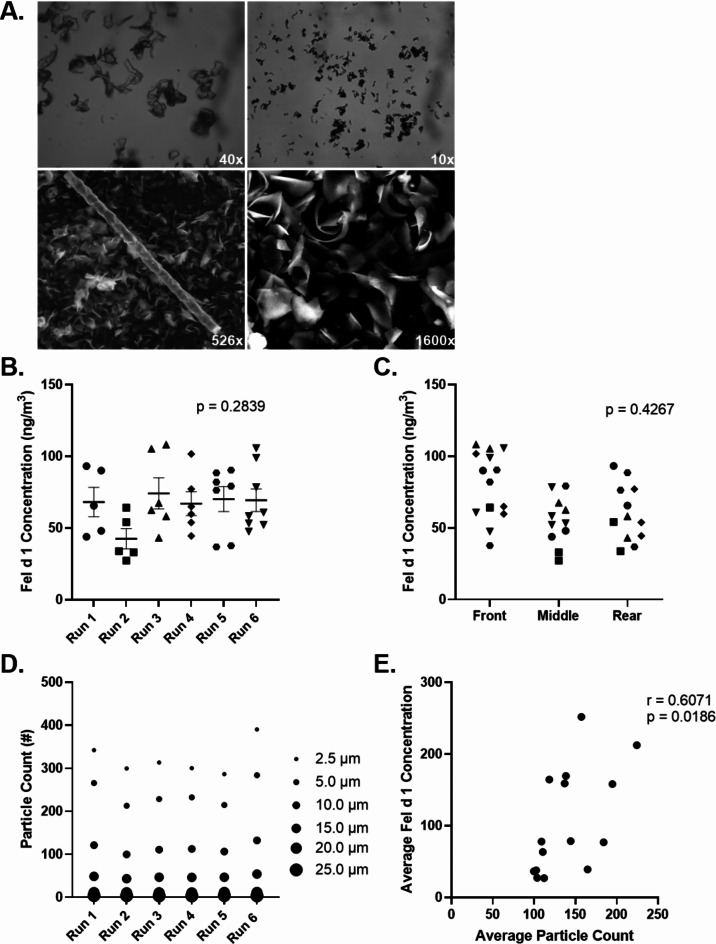
Consistent Fel d 1 concentrations and particle counts were observed across runs in the Specialized Particulate Control Environmental Exposure Unit. Purchased cat dander allergen visualized under light and scanning electron microscopy (**A**) demonstrated a heterogenous product, whereby Fel d 1 cannot be easily visually quantified. This resulted in the use of a Fel d 1-specific ELISA to assess Fel d 1 levels in air samples, collected using sampling cassettes. Across 6 independent trial runs, defined as 2-hour sessions of cat dander distribution in the SPaC-EEU, with similar equipment setups, Fel d 1 concentrations (ng/m^3^) were consistent both overall (**B**) and in various locations of the room (**C**). Particles sized 2.5 μm, captured using a laser particle counter (LPC) were consistently most abundant. A strong, positive correlation (*r* = 0.6071, *p* = 0.0186) between average Fel d 1 concentration and average particle counts (**D**) confirms that the LPC can be used to indirectly track Fel d 1 concentrations in real-time

To assess the distribution of cat dander in the SPaC-EEU, the dander was circulated into the facility using specialized equipment, with the support of variable speed floor and ceiling fans for continuous laminar flow. Consistent with previous technical validations, trial runs were conducted as 2-hour sessions of allergen distribution, with the collection of air samples on open-faced sampling cassettes (Zefon International, USA) and particles (sized 2.5 μm, 5.0 μm, 10.0 μm, 15 μm, 20.0 μm, and 25.0 μm) using a laser particle counter (LPC) located at the centre of the room. Protein was isolated from the sampling cassettes on the day of collection and analysed with a Fel d 1-specific ELISA.

Utilizing similar equipment settings, six independent sessions in the SPaC-EEU showed consistent concentrations of Fel d 1 (*p* = 0.2839; Fig. 1B), with no significant differences (*p* = 0.4267) in concentrations observed between the front, middle, and rear of the room (Fig. 1C). There was also consistency in Fel d 1 distribution between the left, centre, and right locations of the SPaC-EEU (*p* = 0.8205). The most prevalent particle distributed across these runs was consistently the 2.5 μm size (Fig. 1D).

Sampling cassettes allowed us to quantify Fel d 1 concentration at the end of an exposure, whereas the LPC reports particle counts in real-time at 10-second intervals and has historically been used anecdotally to inform concentrations of distributed allergen. Four independent sessions in the SPaC-EEU were conducted, with particle data collected using the LPC and repeated air sampling using cassettes completed at 30- to 120-minute intervals. When comparing average Fel d 1 concentrations quantified from cassettes with average LPC particle counts at corresponding timepoints (Fig. 1E), we observed a significant positive correlation (*r* = 0.6071, *p* = 0.0186). This asserts the capacity for indirect monitoring of Fel d 1 concentrations in real-time using the LPC.

Lastly, we assessed the ability of rotorod impact sampling, which is used for air sampling with pollen allergens, to capture Fel d 1 concentrations. Samples were collected in two SPaC-EEU runs alongside sampling cassettes, and it was found that rotorods captured significantly lower (*p* < 0.05) Fel d 1 than sampling cassettes. This further reinforces the notion that the rotorod impaction method is inferior to cassettes when sampling for perennial allergens.

In summary, this technical validation confirms that the dispersal equipment in the SPaC-EEU can effectively distribute cat allergen throughout the facility in a reliable and consistent manner. The LPC has been proven to be a suitable method for indirect monitoring of real-time Fel d 1 concentrations in the SPaC-EEU, ensuring allergen dispersal consistency over time. Following this successful technical validation, the SPaC-EEU can undergo clinical validation with human participants.

## Data Availability

The data supporting this study are available upon request from the corresponding author.

## Abbreviations

AR: Allergic Rhinitis
EEU: Environmental Exposure Unit
ELISA: Enzyme–Linked Immunosorbent Assay
Fel d 1: Felis domesticus allergen 1
HDM: House Dust Mite
LPC: Laser Particle Counter
SPaC: EEU–Specialized Particulate Control Environmental Exposure Unit
